# Is anti‐viral defence the evolutionary origin of mRNA turnover? (Comment on DOI 10.1002/bies.201600100)

**DOI:** 10.1002/bies.201600140

**Published:** 2016-08-01

**Authors:** Jan Rehwinkel

**Affiliations:** ^1^ Medical Research Council Human Immunology UnitMedical Research Council Weatherall Institute of Molecular MedicineRadcliffe Department of MedicineUniversity of OxfordOxfordUK

In this issue of *BioEssays*, Hamid and Makeyev explore an interesting new idea regarding the evolutionary origins of regulated mRNA turnover pathways 1. One such pathway is nonsense‐mediated decay (NMD) that detects and targets for degradation a subset of mRNAs including transcripts with premature translation termination codons. The authors develop a model in which these pathways initially evolved to target viral RNAs for degradation. They further postulate that these pathways were later re‐purposed to regulate the host cell's gene expression. This hypothesis – that molecular mechanisms nowadays primarily involved in regulated mRNA decay have their origin in anti‐viral defence – provides interesting new perspectives on host‐pathogen co‐evolution and on molecular recognition of RNA.

The hypothesis is supported by recent results that NMD not only regulates cellular transcripts but also viral RNAs 2, 3. NMD recognizes unusual patterns of mRNA translation, particularly atypical positions of stop codons. This feature is shared by some viral mRNAs as a result of the compact genome structure of many viruses and predisposes them to NMD 1, 2, 3. Another example for regulated mRNA decay that also targets viral RNAs is a group of RNA‐binding proteins containing Zn‐fingers that destabilize specific mRNAs. Indeed, one of these proteins, Regnase (also known as Zc3h12a or MCPIP1), not only specifically recognises and degrades cellular mRNAs encoding cytokines but also viral transcripts. Hamid and Makeyev explain such anti‐viral activities of cellular mRNA decay pathways in extant species as evolutionary remnants, and argue that those pathways developed early during eukaryotic evolution to detect and destroy viral RNAs. Indeed, some of the proteins involved in these pathways are broadly conserved, and it is conceivable that early development of eukaryotes was shaped by pressure from RNA pathogens. An interesting question relates to the circumstances that allowed virus‐specific mRNA decay pathways to later shift towards roles in targeting endogenous RNAs without compromising host fitness during infection. The emergence of novel immune defences such as the interferon system may have made such an exaptation possible.

NMD and other regulated mRNA decay pathways need to correctly identify target mRNAs. Interestingly, a similar molecular recognition problem of selecting RNA molecules is faced by the innate immune system. Defence against RNA viruses often involves cellular recognition of viral RNA molecules. This triggers direct effector functions such as degradation or sequestration of viral RNAs as well as the activation of signalling pathways that induce an antiviral state 4, 5. These mechanisms that specifically target or sense viral RNAs must be able to distinguish between “self” and “foreign” RNAs, given that uninfected cells already contain vast quantities of endogenous RNAs. The hypothesis discussed by Hamid and Makeyev suggests that molecular selection of specific RNA molecules has its roots in ancient anti‐viral defence mechanisms. This concept is interesting in that it connects many aspects of mRNA metabolism and innate immunity. It further illustrates the potency of the arms‐race between hosts and pathogens in shaping evolution of new molecular systems.
